# Herd-level prevalence of *Mycoplasma bovis* antibodies in bulk tank milk samples in Austrian dairy herds and risk factors associated with herd seropositive status

**DOI:** 10.1007/s11259-026-11116-4

**Published:** 2026-02-21

**Authors:** Jasmin Laschinger, Helena Wieser, Patricia Hoop, Manolis Lyrakis, Thomas Wittek, Joachim Spergser, Katharina Lichtmannsperger

**Affiliations:** 1 Clinical Department for Farm Animals and Food System Science, Clinical Centre for Ruminant and Camelid Medicine, University of Veterinary Medicine Vienna, Veterinaerplatz 1, Vienna, 1210 Austria; 2 Platform for Bioinformatics and Biostatistics, Department of Biological Sciences and Pathobiology, University of Veterinary Medicine Vienna, Veterinaerplatz 1, Vienna, 1210 Austria; 3 Department of Biological Sciences and Pathobiology, Centre of Pathobiology, University of Veterinary Medicine Vienna, Veterinaerplatz 1, Vienna, 1210 Austria

**Keywords:** Mycoplasma bovis, Antibody, Bulk tank milk, ELISA, ID-screen, Open herd policy, Risk factors

## Abstract

*Mycoplasma bovis* is an emerging pathogen worldwide and is associated with various disease manifestations in cattle of all ages. Several potential herd-level risk factors associated with *M. bovis* positive herd status have been identified in recent years. However, only a few reports of *M. bovis* in Austrian cattle herds are available, and no nationwide prevalence data are available. The objectives of this study were to determine the apparent prevalence of *M. bovis* antibodies in bulk tank milk (BTM) samples from Austrian dairy farms and to identify potential risk factors associated with *M. bovis* antibody-positive herds. In total, 674 BTM samples were collected nationwide and analysed using the ID-Screen^®^ *Mycoplasma bovis* Indirect (IDvet, Grabels, France) ELISA. In addition, farm-specific data and management practices were collected through an accompanying questionnaire to identify potential risk factors for *M. bovis* antibody positivity status. An apparent herd prevalence of 5.2% was observed among Austrian dairy farms. The apparent prevalence across federal states ranged from 0% to 14.8%. Logistic regression analysis revealed a significant association between herd replacement policy (open vs. closed) and herd-level *M. bovis* antibody status (*p* = 0.005), with a closed herd replacement policy associated with a protective effect.

**Supplementary Information:**

The online version contains supplementary material available at 10.1007/s11259-026-11116-4.

## Introduction

*Mycoplasma bovis* is an emerging pathogen associated with pneumonia, otitis media, arthritis, and mastitis in cattle of all ages (Maunsell et al. 2011; Calcutt et al. 2018). This bacterium was first isolated in 1961 in the USA and has since spread globally *via* cattle movements (Su et al. 2025). In Austria, there are few reports on the occurrence of *M. bovis* in cattle (Baumgartner et al. 2006; Spergser et al. 2013; Pothmann et al. 2015). However, there are no comprehensive studies on the prevalence of this bacterium. Diagnosing *M. bovis* can be challenging at both individual animal and herd-level, as the pathogen can lead to various disease manifestations that require different sampling sites and diagnostic tests. In addition, subclinical infections and intermittent shedding can further complicate detection (Maunsell et al. 2011). Herd screening for past exposure can be done by testing bulk tank milk (BTM) samples for antibodies (Parker et al. 2017). The ID Screen^®^*Mycoplasma bovis* indirect ELISA kit (IDvet, Grabels, France) showed the highest diagnostic performance after optimization of cutoff values, with a sensitivity of 89.0% and 93.5%, and specificity of 83.4% and 98.6%, respectively, compared to other commercially available *M. bovis* antibody ELISAs (Andersson et al. 2019; Bokma et al. 2024).

There were several potential herd-level risk factors associated with *M. bovis* positive herds identified, such as introduction of asymptomatically infected animals (Maunsell et al. 2011), farms with a breeding bull and without a calving pen (Gille et al. 2018), high mean milk production, appropriate stimulation until milk-let-down, fore-stripping, animal movements (cattle shows and trade), presence of stress-factors, the use of a specific brand of milking equipment (Aebi et al. 2015), increasing number of lactating cows (Pinho et al. 2013), and others.

The aims of this study were (1) to determine the apparent prevalence of *M. bovis* antibodies in BTM samples from Austrian dairy farms and (2) to identify potential risk factors associated with *M. bovis* antibody-positive herds.

## Materials and methods

### Sample collection

In a previous study, BTM samples from Austrian dairy farms were voluntarily submitted by farmers using 10 millilitre test tubes containing 950 µl of the preservative boric acid (H₃BO₃) for Q fever antibody testing (Throughout Austria, 98 veterinary practices, each covering a broad catchment area, were involved). Additionally, farm-specific data and management practices were collected through a questionnaire. All former study participants were contacted by email or phone to obtain their consent to use their previously submitted BTM sample in the present study, as well as to answer four additional questions about potential *M. bovis* specific risk factors on their farms. All BTM samples were stored at −26 °C at the University of Veterinary Medicine Vienna.

The sample size was calculated using Cochran’s formula with finite population correction, assuming a 95% confidence level (Z = 1.96), an expected prevalence of 30%, and a margin of error of 5%, resulting in a minimum required sample size of approximately 320 farms (Pourhoseingholi et al. 2013; Ahmed 2024), i.e. 1.2% of dairy farms across Austria (Rinderzucht Austria 2024). To improve robustness and regional representativeness, a larger number of samples was targeted. Given the voluntary participation of dairy farmers, sampling of approximately 2–3% of dairy farms per federal state was intended. Therefore, additional BTM samples were collected by cooperating veterinarians in the federal states of Vorarlberg and Tyrol potentially targeting interested farmers or herds with suspected infections. Cooperating veterinarians were provided with sample kits consisting of one 10 millilitre sample tube containing 950 µl of the preservative boric acid (H₃BO₃) from KABE-Labortechnik GmbH, Germany, and a questionnaire with 13 questions, which they distributed to interested farmers. One question concerning housing conditions was excluded from the evaluation post hoc due to imprecise wording. The sample kits were refrigerated and sent by express mail to the University of Veterinary Medicine Vienna, where they were stored at −26 °C until analysis.

The BTM samples analysed in this study were collected between 2019 and 2025 and represented different farms; no farm was sampled more than once. In total, 2.6% (674 of 25,826) of dairy farms in Austria were included in this study. Sampling covered 3.6% (3 of 84) of dairy farms in Burgenland, 3.2% (123 of 3,842) in Styria, 2.8% (149 of 5,295) in Tyrol, 2.7% (107 of 3,987) in Lower Austria, 2.5% (49 of 1,966) in Carinthia, 2.4% (143 of 5,877) in Upper Austria, and 2.1% each in Salzburg and Vorarlberg (73 of 3,467 and 27 of 1,308, respectively).

### Laboratory analysis

In the laboratory of the Clinical Department for Farm Animals and Food System Science, University of Veterinary Medicine Vienna, all 674 BTM samples were tested for the presence of *M. bovis* antibodies using the commercially available ID-Screen^®^ *Mycoplasma bovis* Indirect (IDvet, Grabels, France) ELISA according to the manufacturer’s instructions. The ELISA reactions were read using a photometer (Tecan Sunrise™, Tecan Trading AG, Männedorf, Switzerland) and displayed using software (Magellan™, Tecan Trading AG). The optical density (OD) was measured at 450 nm.

The sample-to-positive percentage (S/P%) for each sample and test was calculated using the following formula:

$$\:S/P\%=\frac{\left(\mathrm{S}\mathrm{a}\mathrm{m}\mathrm{p}\mathrm{l}\mathrm{e}\:\mathrm{o}\mathrm{p}\mathrm{t}\mathrm{i}\mathrm{c}\mathrm{a}\mathrm{l}\:\mathrm{d}\mathrm{e}\mathrm{n}\mathrm{s}\mathrm{i}\mathrm{t}\mathrm{y}\:\right(\mathrm{O}\mathrm{D})\:-\:\mathrm{n}\mathrm{e}\mathrm{g}\mathrm{a}\mathrm{t}\mathrm{i}\mathrm{v}\mathrm{e}\:\mathrm{c}\mathrm{o}\mathrm{n}\mathrm{t}\mathrm{r}\mathrm{o}\mathrm{l}\:\mathrm{O}\mathrm{D})}{(\mathrm{p}\mathrm{o}\mathrm{s}\mathrm{i}\mathrm{t}\mathrm{i}\mathrm{v}\mathrm{e}\:\mathrm{c}\mathrm{o}\mathrm{n}\mathrm{t}\mathrm{r}\mathrm{o}\mathrm{l}\:\mathrm{O}\mathrm{D}\:-\:\mathrm{n}\mathrm{e}\mathrm{g}\mathrm{a}\mathrm{t}\mathrm{i}\mathrm{v}\mathrm{e}\:\mathrm{c}\mathrm{o}\mathrm{n}\mathrm{t}\mathrm{r}\mathrm{o}\mathrm{l}\:\mathrm{O}\mathrm{D})}\times\:100$$

As recommended by the manufacturer, the cutoff for a positive sample was set at S/P% ≥ 30% after the BTM samples were analysed using the overnight incubation protocol.

Positive results were further classified as shown in Table 1.

**Table 1:** Classification of *Mycoplasma bovis* antibody positive bulk tank milk results, using the ID-Screen^®^ *Mycoplasma bovis* Indirect (IDvet, Grabels, France) ELISA, according to the manufacturer. S/P% = sample-to-positive percentage

S/*P*% result	evaluation
30% − 49%	+
50% − 99%	++
100% − 149%	+++
≥ 150%	++++

In addition to positive and negative controls, a cut-off control was included in each analysis as an internal quality assurance measure.

### Statistical analysis

Raw data were assessed for completeness and plausibility. Initial data processing was conducted using Microsoft Excel 2020 (Microsoft Corp., Redmond, WA, USA). The apparent prevalence for Austria and for each federal state was calculated.

Statistical analysis was performed in R (R version 4.5.0) (R Core Team 2025). Significance was declared at 5% cut off. Questions with responses that represent combinations of different possible answers were split into multiple columns (one column per answer with yes/no). Associations between questions were evaluated via Fisher’s exact tests (function *fisher.test*, option ‘simulate.p.value = TRUE’). If a zero was present in the contingency table between two questions, one was added in all the cells of that table. Multiple association tests were corrected for multiple testing using the Bonferroni–Holm method (function *p.adjust*).

The association between each question and *M. bovis* status was evaluated via Fisher’s exact tests (univariate analysis, function *fisher.test*, option ‘simulate.p.value = TRUE’). Questions with a near-complete dataset that showed potential association (*p* < 0.2) were included in a linear regression model to account for potential confounding (function *glm*, option ‘family = binomial(link = “logit”)’). *M. bovis* status was fitted as a binary response. Multicollinearity between the fixed effects of that model (questions) was evaluated via variance-inflation factors (package *car*, version 3.1.3, function *vif*) (Fox and Weisberg 2019). Overall significance for questions with more than two responses was evaluated using a likelihood ratio test via the *anova* function, comparing the full model to a reduced model excluding that question. Pairwise comparisons between the responses of each question were conducted via the estimated marginal means (package *emmeans*, version 1.11.0, function *emmeans*) (Lenth 2025). For presentation, estimated marginal means were back transformed from the *logit* scale (option ‘type = “response”’ in the function *emmeans*). Multiple pairwise comparisons for questions with more than two responses were corrected for multiple testing via the Tukey method.

## Results

A total of 674 BTM samples from the federal states of Lower Austria, Upper Austria, Burgenland, Styria, Carinthia, Salzburg, Tyrol, and Vorarlberg were analysed. The overall apparent prevalence of *M. bovis* antibodies in BTM, as determined by ELISA, was 5.2% for Austria. The apparent prevalence in each federal state ranged from 0% to 14.8% (Table 2). These estimates did not differ significantly (*p* = 0.49). Among the 35 farms that tested positive by ELISA, 16 were classified as ‘+’ (low positive), 11 as ‘++’ (moderate positive), 4 as ‘+++’ (strong positive), and 4 as ‘++++’ (very strong positive), according to the test manufacturer’s scoring criteria.

**Table 2:** Overview of the bulk tank milk (BTM) samples analysed and the number and proportion of ELISA-positive results for *Mycoplasma bovis* antibodies, stratified by federal state of Austria

Federal state	BTM samples analysed	BTM samples with positive ELISA for *M. bovis* antibodies	Prevalence in %
Lower Austria	107	7	6.5
Carinthia	49	2	4.1
Burgenland	3	0	0.0
Styria	123	4	3.3
Vorarlberg	27	4	14.8
Tyrol	149	8	5.4
Salzburg	73	3	4.1
Upper Austria	143	7	4.9

There were positive associations between Vorarlberg and the breed Brown Swiss (*p* < 0.001), between Tyrol and the breed Tyrolean Grey (*p* < 0.001), between Salzburg and the Pinzgauer cattle breed (*p* = 0.002), as well as between Upper Austria and Fleckvieh cattle (*p* < 0.001). A negative association between Vorarlberg and cattle of the breed Fleckvieh was also observed (*p* < 0.001).

Herd size, annual herd-level milk yield, herd replacement policy (open vs. closed), location in the federal state of Vorarlberg, and the breeds Brown Swiss, Holstein-Frisian, and Jersey showed potential association with herd-level *M. bovis* antibody status in the univariate analysis (*p* < 0.2) (Table 3 and Supplementary Table 4).

**Table 3:** Questionnaire provided to the farmers to evaluate internal and external biosecurity and general herd management displaying only the subset of questions with a near-complete dataset, that were included in the logistic regression (multivariate) analysis and are marked with a plus sign (+). The number of responses with negative and positive *Mycoplasma bovis* antibody ELISA results is provided. Questions for which the association with herd-level *M. bovis* antibody status yielded a *p*-value up to 0.2 in fisher’s exact test are marked with an asterisk (*) and the corresponding *p*-value is reported inside parentheses

Question and answers	Negative	Positive	Total responses (out of 674)
How many cattle are kept on farm? *^(*p*=0.011)+^			673
< 50 50–80 80–120 > 120	477 126 23 12	19 10 5 1	
Predominant breed on farm?			671
Fleckvieh Brown Swiss*^(*p*=0.18)+^ Holstein-Frisian*^(*p*=0.058)+^ other breed (e.g. Jersey*^(*p*=0.13)+^) heterogeneous breed composition	450 45 47 20 74	21 2 3 1 8	
Annual herd milk yield? *^(*p*=0.008)+^			673
< 8,000 L 8,000–9,000 L > 10,000 L	298 221 119	8 20 7	
Open herd policy, i.e. purchasing cattle from other farms? *^(*p*=0.006)+^			674
Yes No	216 423	20 15	
Federal state of the farm^1^			674
Lower Austria Carinthia Burgenland Styria Vorarlberg*^(*p*=0.046)+^ Tyrol Salzburg Upper Austria	100 47 3 119 23 141 70 136	7 2 0 4 4 8 3 7	

^1^Vienna was excluded from the analysis because just 5 dairy cows are kept in this federal state (Rinderzucht Austria 2024).

The logistic regression showed a significant association between herd replacement policy (open vs. closed) and herd-level *M. bovis* antibody status (*p* = 0.005). The probability for a positive *M. bovis* antibody ELISA result was higher for farms with an open herd policy (OR = 0.363; 95% CI 0.178–0.741). In addition, there was a trend between the annual herd-level milk yield and herd-level *M. bovis* antibody status (*p* = 0.051), with a larger probability for a positive *M. bovis* antibody ELISA for farms with intermediate annual herd-level milk yield (8,000–9,000 L) compared to farms with a low annual herd-level milk yield (< 8,000 L) (OR = 0.345; 95% CI 0.14–0.853; *p* = 0.055).

## Discussion

A total of 35 of the 674 BTM samples tested positive for *M. bovis* antibodies, resulting in an apparent herd prevalence of 5.2% in Austrian dairy herds. Each ELISA analysis included positive and negative controls as well as a cut-off control as internal quality assurance measures, ensuring the reliability of the test results. Because participation in the present study was voluntary, we relied on the cooperation of participating veterinarians and farmers, which may have resulted in a non-representative sample composition. Results should therefore be interpreted with caution when extrapolating to nationwide prevalence estimates. Nevertheless, this study provides valuable insights into *M. bovis* occurrence across multiple regions throughout Austria. Herd-level *M. bovis* prevalence in Europe, determined using various diagnostic techniques, i.e., ELISA, culture, or PCR, applied to BTM samples over the last two decades, has ranged from 0% to 45%. For instance, in Ireland, an apparent prevalence of 45% was detected in BTM samples using antibody ELISA, while in Belgium, 7.1% and 24.8% were detected in BTM samples by using PCR and ELISA serology, respectively (Gille et al. 2018; McAloon et al. 2022).

The apparent prevalence among the individual federal states varied considerably in our study, ranging from 0% to 14.8%. This variation may be partly attributable to the relatively small sample sizes in some federal states, which can lead to disproportionately high percentage estimates when only a few positive cases are detected. Burgenland was represented by only three farms (3.6% of dairy farms in this federal state), which is likely insufficient to provide reliable prevalence estimates and should be acknowledged as a limitation. However, Burgenland is a small federal state with low number of dairy farms. Another potentially influencing factor is the timing of sampling relative to the infection status, as the incubation period of *M. bovis* varies (Calcutt et al. 2018), and an immunological test is only effective once an animal seroconverts. However, individual animal antibody titers correlate poorly with clinical disease or infection status, as not all infected animals develop high titers and elevated titers can persist for months (Maunsell et al. 2011). At the group level, in contrast, seroconversion or high antibody titers are predictive of active *M. bovis* infection. Serology is therefore most appropriately used as a herd-level screening tool (Maunsell et al. 2011). Despite the significant association between the BTM ELISA optical density coefficient and the within-herd seroprevalence (Parker et al. 2017), there is currently no reliable source that specifies a defined minimum prevalence of *M. bovis* antibody-positive cows in the herd as a prerequisite for a positive BTM ELISA. The outcome of a BTM *M. bovis* antibody ELISA is contingent on numerous factors, including the time elapsed since the initial outbreak of *M. bovis* infection in the herd (Parker et al. 2017).

BTM *M. bovis* antibody ELISA testing is suitable for identifying *M. bovis*-infected mammary glands (Maunsell et al. 2011). However, mastitic milk is typically withheld from the bulk tank, and in our study, only cows contributing to the bulk tank were tested. This might have led to an underestimation of the true prevalence. Moreover, it should be critically considered that some samples were stored at −26 °C for several years prior to analysis. The literature reports inconsistent findings on the effect of long-term freezing of colostrum on immunoglobulin concentration, with both modest declines and reasonable preservation described (Stalker et al. 2023; Westhoff and Mann 2025). However, the effect of long-term frozen storage on specific immunoglobulin in mature milk is not well established and may lead to degradation and false-negative results. Storage-time bias cannot be ruled out in our study. We therefore emphasize that the calculated prevalence represents an apparent prevalence under the given sampling and storage conditions.

The higher prevalence of positive herds in the western part of Austria may be explained by the fact that most samples from this region were collected specifically for the purpose of this study, potentially targeting herds with suspected infections, or due to the intensive use of communal alpine pastures in this region and the consequent mixing of cattle from different farms. Biosecurity-related practices such as communal grazing or foot trimming should be examined in future studies as possible risk factors for *M. bovis* antibody-positive status in dairy herds. Additionally, the higher prevalence could potentially be linked to a previous outbreak, re-emergence, and subsequent spread of a single endemic *M. bovis* strain in the Austrian Alps (Spergser et al. 2013), representing a hypothesis for future research.

Of the 674 BTM samples analyzed, 70.2% (471 of 671 BTM samples; 3 missing responses) originated from farms predominantly keeping cattle of the breed Fleckvieh. The remaining farms mainly kept Holstein-Friesian (7.5%), Brown Swiss (7%), other breeds like Jersey or Pinzgauer (3.1%), or a mix of various breeds (12.2%) (Table 3). Given the limited variability, breed-related comparisons should be interpreted cautiously.

The response rate to the survey was partially very low, especially to the additional questions about potential *M. bovis* specific risk factors on the farms, which limited the possibility of performing a robust statistical analysis. Analysing risk factors according to the strength of positivity could provide additional insights. However, due to the overall low number of positive samples, such an analysis was considered statistically not meaningful in our dataset, but it represents an important consideration for future research. Nevertheless, a closed herd policy was identified as a protective factor, which is in accordance with previous studies (Maunsell et al. 2011; Aebi et al. 2015; Calcutt et al. 2018). A higher probability of a positive *M. bovis* antibody ELISA was observed on farms with an intermediate annual herd-level milk yield. This finding contradicts previous literature, which often associates high average milk production with an increased risk (Aebi et al. 2015; Ninković et al. 2024). We hypothesize that this discrepancy is mainly due to the high proportion (70.2%) of dairy farms keeping predominantly cattle of the breed Fleckvieh in our study. As a dual-purpose breed, Fleckvieh has lower milk yield than specialized dairy cattle breed, which were predominantly included in previous studies. Furthermore, the thresholds used to define low, intermediate, and high annual herd-level milk yield vary between studies, which may also have influenced the observed results.

## Conclusion

*M. bovis* BTM antibodies were detected widely across Austrian dairy farms. Therefore, this pathogen should be included in routine diagnostics for *M. bovis*-associated disease manifestations in Austrian cattle, especially in dairy cows with mastitis or calves with pneumonia, arthritis or otitis media. Since an open herd policy has been identified as a risk factor for herd seropositivity, farms should pay closer attention to biosecurity to prevent the introduction and spread of this bacterium.

## Supplementary Information

Below is the link to the electronic supplementary material.

MOESM1 (DOCX 17.8 KB)

## Data Availability

The datasets generated and analysed during the current study are available from the corresponding authors on reasonable request.
