# Supplementary material for: Herd-level prevalence of Mycoplasma bovis antibodies in bulk tank milk samples in Austrian dairy herds and risk factors associated with herd seropositive status
Source: Vet Res Commun. 2026 Feb 21;50(2):165. doi: 10.1007/s11259-026-11116-4 (PMC12924781; doi:10.1007/s11259-026-11116-4)
Supplement: Supplementary file 1 — (DOCX 17.8 KB) [file 11259_2026_11116_MOESM1_ESM.docx]

**Supplementary Material**

Supplementary Table 4: Questionnaire provided to the farmers to evaluate internal and external biosecurity and general herd management. The number of responses with negative and positive *Mycoplasma bovis* antibody ELISA results is provided. Questions for which the association with herd-level *M. bovis* antibody status yielded a *p*-value up to 0.2 in Fisher’s exact test are marked with an asterisk (*) and the corresponding *p*-value is reported inside parentheses. A subset of these questions, with a near-complete dataset, was included in the logistic regression (multivariate) analysis and is marked with a plus sign (+).

| **Question and answers** | **Negative** | **Positive** | **Total responses (out of 674)** |
| --- | --- | --- | --- |
| How many cattle are kept on farm?*^(^*^p^*^=0.011)+^ |  |  | 673 |
| <50 50-80 80-120 >120 | 477 126 23 12 | 19 10 5 1 |  |
| Predominant breed on farm? |  |  | 671 |
| Fleckvieh Brown Swiss*^(^*^p^*^=0.18)+^ Holstein-Frisian*^(^*^p^*^=0.058)+^ other breed (e.g. Jersey*^(^*^p^*^=0.13)+^) heterogeneous breed composition | 450 45 47 20 74 | 21 2 3 1 8 |  |
| Annual herd milk yield?*^(^*^p^*^=0.008)+^ |  |  | 673 |
| <8,000 litres 8,000-9,000 litres >10,000 litres | 298 221 119 | 8 20 7 |  |
| Dairy cows and youngstock kept in the same building? | |  | 674 |
| Yes No | 517 122 | 29 6 |  |
| Open herd policy, i.e. purchasing cattle from other farms?*^(^*^p^*^=0.006)+^ | |  | 674 |
| Yes No | 216 423 | 20 15 |  |
| From how many different farms are animals purchased each year?*^(^*^p^*^=0.14)^ | | | 233 |
| <5 5-15 >15 | 193 18 1 | 18 2 1 |  |
| Where are the animals purchased from? |  |  | 243 |
| Same federal state different federal state*^(^*^p^*^=0.08)^ outside Austria combination of above answers | 183 22 1 16 | 14 4 0 3 |  |
| Are calves vaccinated regularly against respiratory diseases?*^(^*^p^*^=0.18)^ | | | 191 |
| Yes No | 8 168 | 2 13 |  |
| Federal state of the farm^1^ |  |  | 674 |
| Lower Austria Carinthia Burgenland Styria Vorarlberg*^(^*^p^*^=0.046)+^ Tyrol Salzburg Upper Austria | 100 47 3 119 23 141 70 136 | 7 2 0 4 4 8 3 7 |  |
| Known history of *Mycoplasma bovis* on farm? |  |  | 191 |
| Yes No | 4 172 | 1 14 |  |
| Is there an increased incidence of the following diseases in cattle on the farm? | | | 191 |
| Respiratory diseases (Yes \| No) Otitis media in calves (Yes \| No) Arthritis (Yes \| No) Mastitis with negative bacteriological findings (Yes \| No) None of the options (Yes \| No) | 45 \| 131 2 \| 174 6 \| 170 34 \| 142  72 \| 104 | 2 \| 13 0 \| 15 0 \| 15 3 \| 12  5 \| 10 |  |
| Feeding of milk with elevated somatic cell count to calves? | | | 191 |
| No Yes, to male and female calves Yes, to male calves only Yes, to female calves only | 40 80 56 0 | 2 8 5 0 |  |

^1^Vienna was excluded from the analysis because just 5 dairy cows are kept in this federal state (Rinderzucht Austria 2024).
